# Higher sterol content regulated by *CYP51* with concomitant lower phospholipid content in membranes is a common strategy for aluminium tolerance in several plant species

**DOI:** 10.1093/jxb/eru455

**Published:** 2014-11-21

**Authors:** Tadao Wagatsuma, Md. Shahadat Hossain Khan, Toshihiro Watanabe, Eriko Maejima, Hitoshi Sekimoto, Takao Yokota, Takeshi Nakano, Tomonobu Toyomasu, Keitaro Tawaraya, Hiroyuki Koyama, Matsuo Uemura, Satoru Ishikawa, Takashi Ikka, Akifumi Ishikawa, Takeshi Kawamura, Satoshi Murakami, Nozomi Ueki, Asami Umetsu, Takayuki Kannari

**Affiliations:** ^1^Faculty of Agriculture, Yamagata University, Tsuruoka 997-8555, Japan; ^2^HMD Science and Technology University, Dinajpur, Bangladesh; ^3^Graduate School of Agriculture, Hokkaido University, Sapporo 060-8589, Japan; ^4^Faculty of Agriculture, Utsunomiya University, Utsunomiya 321-8505, Japan; ^5^Department of Bioscience, Teikyo University, Utsunomiya 320-8551, Japan; ^6^Antibiotics Laboratory RIKEN, Wako, Saitama 351-0198, Japan; RIKEN Centre for Sustainable Resource Science, Wako, Saitama 351-0198, Japan; CREST, Japan Science and Technology Agency (JST), Japan; ^7^Faculty of Agriculture, Yamagata University, Tsuruoka 997-8555, Japan; ^8^Faculty of Applied Biological Sciences, Gifu University, Gifu 501-1193,Japan; ^9^Cryobiosystem Research Centre, Faculty of Agriculture, Iwate University, Morioka 020-8550, Japan; ^10^National Institute for Agro-Environmental Science, Tsukuba 305-8604, Japan; ^11^Faculty of Agriculture, Shizuoka University, Shizuoka 422-8529, Japan

**Keywords:** Aluminium (Al) tolerance, *CYP51*, phospholipid, plasma membrane, sterol, uniconazole-P.

## Abstract

Several studies have shown that differences in lipid composition and in the lipid biosynthetic pathway affect the aluminium (Al) tolerance of plants, but little is known about the molecular mechanisms underlying these differences. Phospholipids create a negative charge at the surface of the plasma membrane and enhance Al sensitivity as a result of the accumulation of positively charged Al^3+^ ions. The phospholipids will be balanced by other electrically neutral lipids, such as sterols. In the present research, Al tolerance was compared among pea (*Pisum sativum*) genotypes. Compared with Al-tolerant genotypes, the Al-sensitive genotype accumulated more Al in the root tip, had a less intact plasma membrane, and showed a lower expression level of *PsCYP51*, which encodes obtusifoliol-14α-demethylase (OBT 14DM), a key sterol biosynthetic enzyme. The ratio of phospholipids to sterols was higher in the sensitive genotype than in the tolerant genotypes, suggesting that the sterol biosynthetic pathway plays an important role in Al tolerance. Consistent with this idea, a transgenic *Arabidopsis thaliana* line with knocked-down *AtCYP51* expression showed an Al-sensitive phenotype. Uniconazole-P, an inhibitor of OBT 14DM, suppressed the Al tolerance of Al-tolerant genotypes of maize (*Zea mays*), sorghum (*Sorghum bicolor*), rice (*Oryza sativa*), wheat (*Triticum aestivum*), and triticale (×*Triticosecale* Wittmark cv. Currency). These results suggest that increased sterol content, regulated by *CYP51*, with concomitant lower phospholipid content in the root tip, results in lower negativity of the plasma membrane. This appears to be a common strategy for Al tolerance among several plant species.

## Highlight

Higher sterol content regulated by *CYP51* with concomitant lower phospholipid contents in root tips results in higher aluminium tolerance. This strategy is common to different varieties of plant species.

## Introduction

Aluminium (Al) toxicity has been accepted to be a primary and common factor that negatively affects plant growth in acid soils. Improving the Al tolerance of crop plants would be one approach for producing sufficient foods for world agriculture. The results of many physiological studies have suggested that plants have developed various strategies of Al tolerance, including organic acid anion (OA) excretion from roots, modification of cell wall structure (Yang *et al.*, 2010), and Al sequestration (reviewed by Horst *et al.*, 2010; Magalhães, 2010; Ryan *et al.*, 2011). Understanding the molecular mechanisms of these Al-tolerance strategies is essential for establishing efficient breeding programmes, e.g. using marker-assisted selection.

Recent progress in molecular genetics and physiological research has identified various genes that regulate Al tolerance. In particular, genes regulating Al-activated/induced OA excretion have been isolated from various plant species. *ALMT1* (*AL* activated *M*alate *T*ransporter *1*) was first isolated from wheat (*Triticum aestivum*) (Sasaki *et al.*, 2004), while *MATE* (*M*ultidrug *A*nd *T*oxic Compound *E*xtrusion), which is involved in citrate transport, was isolated from sorghum (*Sorghum bicolor*) (Magalhães *et al.*, 2007). Functional orthologues have been characterized in various other plants species, such as *AtALMT1* (Hoekenga *et al.*, 2006) and *AtMATE* (Liu *et al.*, 2009) in *Arabidopsis thaliana*. An Al-tolerant bread wheat genotype derived from a Portuguese landrace exhibited very high basal transcript levels of both citrate and malate transporter genes, *TaMATE1* and *TaALMT1*, respectively (Garcia-Oliveira *et al.*, 2014). Additionally, studies on mutants have identified transcription factors that regulate the expression of these genes, including STOP1 (Sensitive TO Proton Rhizotoxicity *1*) in *Arabidopsis* (Iuchi *et al.*, 2007) and its homologue in rice (*Oryza sativa*), ART1 (*A*luminium *R*esistance *T*ranscription Factor *1*) (Yamaji *et al.*, 2009). Further research has identified that STOP1/ART1 regulate multiple Al-tolerance genes (Sawaki *et al.*, 2009), including *ALS3*/*STAR2* (*AL*uminium *S*ensitive *3* in *Arabidopsis*/*S*ensitive *T*o *A*l *R*hizotoxicity *2*), encoding a prokaryotic-type ABC transporter, *OsMGT1* (*Oryza sativa M*A*G*NESIUM *T*RANSPORTER*1*), encoding a magnesium transporter (Chen *et al.*, 2012), and other genes (Xia *et al.*, 2013). The STOP1/ART1 system is probably conserved among land-plant species, including bryophytes (Ohyama *et al.*, 2013), while several other genes that regulate Al tolerance do not belong to this system. Although studies using Al-sensitive/tolerant mutants have identified several genes regulating Al tolerance (e.g. SLOW WALKER 2) (Nezames *et al.*, 2013), it is likely that many genes have not been identified because of the complexity of the mechanisms of Al tolerance and toxicity.

The physico-chemical and physiological properties of the plasma membrane affect Al tolerance in plants. In previous studies, loss of integrity of the plasma membrane was strongly correlated with the degree of Al damage in the sensitive cultivars after an Al treatment (Ishikawa and Wagatsuma, 1998; Wagatsuma *et al.*, 2005). The lipid composition of the plasma membrane could explain the different responses of plasma membrane integrity to Al between Al-tolerant and Al-sensitive cultivars. Ryan *et al.* (2007) showed that modifying the plasma membrane lipid composition [i.e. a higher Δ^8^-sphingolipid content, and predominance of the (Z)-isomer] conferred Al tolerance in transgenic *Arabidopsis*. The (Z)-isomer of sphingolipids affects the fluidity of the plasma membrane and may alter the raft structure at the plasma membrane. The phospholipid contents in the plasma membrane also affect Al tolerance, which can be explained by the toxic mechanisms of Al^3+^ in solution (Deleers *et al.*, 1985). In a previous study on *Arabidopsis*, simulations of Al toxicity in a complex solution and calculations of the amount of Al^3+^ at the plasma membrane surface based on GEOCHEM-EZ (Shaff *et al.*, 2010) and a speciation-based Gouy-Chapman-Stern model (SGCS) (Kinraide and Wang, 2010) identified that Al^3+^ attracted to the plasma membrane surface determined the degree of Al toxicity (Kobayashi *et al.*, 2013). Because phospholipids are responsible for the negative charge at the plasma membrane surface, the *pah1pah2* (*phosphatidate phosphohydrolase 1* and *2*) mutant, which accumulates phospholipids under P-starved conditions as a result of defective P-recycling, was more Al-sensitive than the wild type under P-stressed conditions. These studies have highlighted the importance of plasma membrane lipid composition in Al tolerance. Zhang *et al.* (1996) reported that the ratio of total sterols to phospholipids in microsomal membranes isolated from 5-mm root tips was slightly higher in an Al-resistant wheat cultivar than in an Al-sensitive one. This finding provided further evidence that the phospholipid contents of the plasma membrane are an important factor in Al tolerance.

Khan *et al.* (2009) reported that Al tolerance was positively correlated with the ratio of sterols to phospholipids in root-tip cells of various rice cultivars. Application of uniconazole-P, an inhibitor of obtusifoliol-14α-demethylase (OBT 14DM), decreased the sterol content in root-tip cells of rice. Uniconazole-P increased the phospholipid to sterol ratio and induced Al sensitivity in an Al-tolerant cultivar. It has been suggested that *CYP51*, which encodes OBT 14DM, has a role in Al tolerance. In the present study, this model was tested by comparing the phospholipid to sterol ratios among various plant species. Molecular cloning and expression analyses showed that expression levels of *CYP51* were lower in an Al-sensitive mutant line of pea than in an Al-tolerant cultivar. Finally, the model was tested using transgenic *Arabidopsis* with knocked-down *CYP51* expression. The results of all of these analyses fitted the model, and strongly suggested that *CYP51* plays a significant role in Al tolerance.

## Materials and methods

### Plant materials and growth conditions

The whole experiment consisted of three parts using different plant materials: three cultivars and one mutant of pea; the wild type and a transformant of *Arabidopsis thalina*; and five plant species including six cultivars and four lines. Seeds of the gibberellin (GA) mutant line and the wild type of pea (*lh* and Torsdag, respectively) were harvested from the Research Farm of Teikyo University, Japan. The *AtCYP51*-KD transgenic line-1 of *Arabidopsis*, which was transformed by Kushiro *et al.* (2001), was used in the present experiments. The seed progenies were obtained using the single-seed descent method. Germination and preculturing of *Arabidopsis* was carried out as described by Toda *et al.* (1999). To collect *Arabidopsis* seeds for T3 progeny, seeds were sown one by one using a pipetter and germinated on Rockfiber (Nittobo Co. Ltd, Tokyo, Japan). The seedlings were fertilized with a 1/1000 dilution of HYPONeX nutrient solution (HYPONeX Japan Ltd, Osaka, Japan) and were grown for 1 week at 22±1 °C under a 12-h light/12-h dark photoperiod. Each 1-week-old seedling was transferred from the Rockfiber to a pot filled with fertilized and sterilized peat soil (Supermix, Sakata Seeds, Yokohama, Japan). Seedlings were watered for 1 week and thereafter grown independently and covered with a transparent plastic cylinder to avoid cross-pollination. Seedlings were fertilized once weekly with 1/1000 diluted HYPONeX nutrient solution and grown under the same light conditions as those described above. Seeds were collected 3 months after germination (Supplementary Figure S1).

The *Arabidopsis* seeds collected were surface sterilized with 1% NaClO, and then kept at 4°C for 3–4 days before planting to synchronize germination. The germinated seeds were transferred to floats for experiments. Each float consisted of a nylon mesh (50 mesh per inch) supported on a plastic photo slide mount. Approximately 20 seeds were placed on each float, and 30 floats were floated on 6 l nutrient solution in the same plastic container (Kobayashi *et al.*, 2005). The basic nutrient solution consisted of 200 µM CaCl_2_, 60 µM MgSO_4_, and other MGRL nutrients (Fujiwara *et al.*, 1992), without inorganic phosphate (Pi), at a strength of 1/50.

The T3 progeny were used for experiments on Al tolerance, visualization of Al accumulation and plasma membrane permeability, and analyses of phospholipids and sterols. Three lines of T3 progeny were used for real-time, quantitative reverse transcription PCR (qRT-PCR) and OA analyses. Seeds of two pea cultivars (*Pisum sativum* L. cv. Harunoka and cv. Hyougo), two sorghum cultivars (*Sorghum bicolor* Moench cv. Super sugar and cv. Kaneko-hybrid), and two maize cultivars (*Zea mays* L. cv. KD 850 and cv. KD 520) were purchased from Kaneko Seeds (Gunma, Japan) and Takii Seeds (Kyoto, Japan). Seeds of two lines of triticale (×*Triticosecale* Wittmark cv. Currency lines ST2 and ST22), two lines of wheat (*Triticum aestivum* L. lines ET8 and ES8), and two cultivars of rice (*Oryza sativa* L. cv. Rikuu-132 and cv. Rikuu-20) were harvested from the Field Science Centre of Yamagata University, Japan. Seeds of pea, sorghum, maize, triticale, wheat, and rice were soaked in tap water under aeration for 24h at 27°C in a growth room and germinated under fluorescent white light (80.7 µmol m^–2^ s^–1^). The germinated seeds were spread on a nylon screen and placed on a container filled with 9 l tap water containing (in mg L^–1^) Ca 8.0, Mg 2.92, K 1.95, and other minor quantities of minerals (P, Fe, Mn, Zn, and Cu) (Akhter *et al.*, 2009).

### Screening for Al tolerance in the presence or absence of a sterol metabolism inhibitor

Screening for Al tolerance was carried out as described by Khan *et al.* (2009). Briefly, roots of young seedlings with a primary root length of ~4cm were pre-treated with 0.2mM CaCl_2_ (pH 4.9) for 6h to allow them to adapt to low-pH conditions. Then, the roots were treated with 0.2mM CaCl_2_ with AlCl_3_ (Al treatment) or without AlCl_3_ (control) at pH 4.9 (or at pH 5.0 for sorghum only) for 24h in the long-term experiments. The concentration of AlCl_3_ was 20 µM for pea, triticale, and maize, 10 µM for rice, 5 µM for wheat, and 2.5 µM for sorghum. In the short-term experiments, the roots of the wild type and the GA mutant of pea were treated for 1h with 0.2mM CaCl_2_ with or without Al in the absence of uniconazole-P [(E)-(S)-1-(4- chlorophenyl)-4,4-dimethyl-2-(1H-1,2,4-triazol-1-yl) pent-1-en-3-ol] (Wako Pure Chemicals, Osaka, Japan). Then, the roots were immersed in 0.2mM CaCl_2_ (pH 5.2) for 9h to allow re-elongation. Consequently, the duration of the Al treatment was 1h, but the duration of the whole treatment was 10h. Root lengths were measured with a ruler. In the long-term experiments, Al tolerance was calculated as the ratio of root elongation of the longest primary root after the 24-h Al treatment to that in the control. In the short-term experiments, Al tolerance was calculated as the ratio of root elongation of the longest primary root after 1-h treatment with AlCl_3_ followed by 9-h re-elongation in 0.2mM CaCl_2_ to that in the control (10-h in 0.2mM CaCl_2_ solution). Twelve seedlings were used for each set of experiments. Data shown are averages of triplicate sets. For the inhibitor experiment, Tween-20 (used to solubilize uniconazole-P) was added to control and Al solutions to a final concentration of 0.0005% (w/v). This concentration of Tween-20 did not inhibit normal root elongation (data not shown). The Al tolerance in the presence of uniconazole-P was calculated by comparing root elongation under (AlCl_3_＋CaCl_2_＋uniconazole-P) conditions to that under (CaCl_2_＋uniconazole-P) conditions. The concentration of uniconazole-P that inhibited root elongation differed among plant species, but not between cultivars or lines (data not shown). Based on preliminary experiments to identify the concentration of uniconazole-P that did not inhibit normal elongation of roots in control solution (data not shown), 0.51 µM uniconazole-P was used for wheat and rice, and 1.02 µM uniconazole-P was used for triticale, maize, and sorghum.

### Screening of Al tolerance and estimation of root growth of *Arabidopsis*

These experiments were conducted as described by Ikka *et al.* (2007) with minor modifications. Briefly, seeds were preincubated at 4°C for 3 days to allow synchronous germination. The germinated seeds were grown on floats on solution with or without 4 µM AlCl_3_ (pH 5.0) under a 12-h light/12-h dark photoperiod (22±1°C). Root lengths were measured on day 7. Images of roots were acquired using a digital camera (SP-350; Olympus, Tokyo, Japan) attached to a stereoscopic microscope (SZ61; Olympus). Then, the lengths of all roots in the image were measured using Image J software. Ten seedlings were used for each measurement, and this process was repeated three times. Al tolerance was calculated as the ratio of root length in the Al treatment to that in the control.

### Visualization of Al accumulation and plasma membrane permeability

After treatment with or without Al for 24h (in the case of pea only, the duration of Al treatment was 1h), whole roots were stained with haematoxylin (0.2% in 0.02% sodium iodide, w/w; pH 4.8) for 15min as described by Polle *et al.* (1978). Al accumulation in the tip portion was observed by stereoscopy (SMZ-10, Nikon, Tokyo, Japan; or SZ-61, Olympus), and Al distribution in a section 2–3mm from the root apex was observed by light microscopy (LABOPHOT; Nikon) (Ishikawa *et al.*, 1996). To visualize plasma membrane permeability, whole roots were stained with fluorescein diacetate-propidium iodide (FDA-PI) (12.5mg l^–1^ FDA, 5mg l^–1^ PI) as described by Ishikawa *et al.* (2001). In the case of pea only, FDA-PI staining was carried out using the following two root samples: (1) a root sample after Al treatment, and (2) a root sample after a 1-h Al treatment followed by a 3-h post-treatment with 0.2mM CaCl_2_. The stained roots were observed under a fluorescence microscope equipped with a B2 filter (excitation filter, 450–490nm; barrier filter, 520nm) (EFDA-2, Nikon).

### Isolation of a full-length cDNA encoding CYP51 in pea

RT-PCR was carried out using an Expand HF PCR system (Roche, Penzberg, Germany) and the primers were degenerated to clone *CYP51* from pea. Pooled cDNA (10ng) prepared from frozen 1-cm root tips of 5-day-old pea seedlings was used as the template. The nucleotide sequences of the degenerate primers were as follows: 5’- TTYAAYGTNCCNACNTTYGG-3’ (sense) and 5’-CCNACNACCAWNGCNTTCCA-3’ (antisense). To determine the full-length cDNA sequence, 5’- and 3’-rapid amplification of cDNA ends (RACE) was performed as described previously (Toyomasu *et al.*, 1998).

### Real-time qRT-PCR

Total RNA was extracted from frozen 1-cm root tips of 5-day-old pea seedlings using an RNAqueous column with Plant RNA Isolation Aid (Ambion, Austin, TX, USA). cDNA was synthesized from 1 µg total RNA with a QuantiTect reverse transcription kit (Qiagen, Hilden, Germany). Real-time qRT-PCR using SYBR Green I was carried out using a TP800 thermal cycler (Takara Bio, Shiga, Japan) as described previously (Sawada *et al.*, 2008) using gene-specific primers. The mean expression level of two replicates was normalized to that of 18S rRNA as the internal control. Total RNA was extracted from *Arabidopsis* by the method of Suzuki *et al.* (2004) using root tissues of plants grown hydroponically in modified MGRL medium for 1 week in the presence of 4 µM AlCl_3_, as described previously (Kobayashi *et al.*, 2007). Transcript levels of *CYP51G1* and *UBQ1* were quantified by real-time PCR using an Applied Biosystems 7300 instrument (Applied Biosystems, Foster City, CA, USA) and specific primer pairs, following the manufacturer’s instructions.

### Malate collection and analysis

The *Arabidopsis* plants used in experiments on malate excretion were grown and treated as described previously (Kobayashi *et al.*, 2007). Briefly, the roots of *in vitro*-grown seedlings were exposed to 0.2mM CaCl_2_ with or without 10 µM AlCl_3_ at pH 5.0 for 24h. Malate was quantified by the enzymatic cycling technique described by Hampp *et al.* (1984).

### Extraction and analysis of phospholipids and sterols

Lipids were extracted and analysed as described previously (Khan *et al.*, 2009). Briefly, lipids were extracted from 10-mm apical root samples. The modified Bligh and Dyer method (1959) (Uemura and Yoshida, 1984) was used to extract phospholipids [isopropanol: chloroform: H_2_O (1:1:1, v/v/v)], and a modified method of Hartmann and Benveniste (1987) was used to extract sterols [dichloromethane: methanol (2:1, v/v)]. Lipids were extracted form whole plants of *Arabidopsis*. After purification and dehydration of the extract, P was analysed by the molybdenum blue method and Δ^5^-sterols were analysed using a spectrophotometric method (Zlatkis and Zak, 1969). For sterols, A_550_ of the samples was measured, and the data are expressed as β-sitosterol (Wako Pure Chemicals) equivalents.

## Results

### Al tolerance, Al accumulation, and plasma membrane permeability of pea cultivars and a pea mutant

**Fig. 1.**
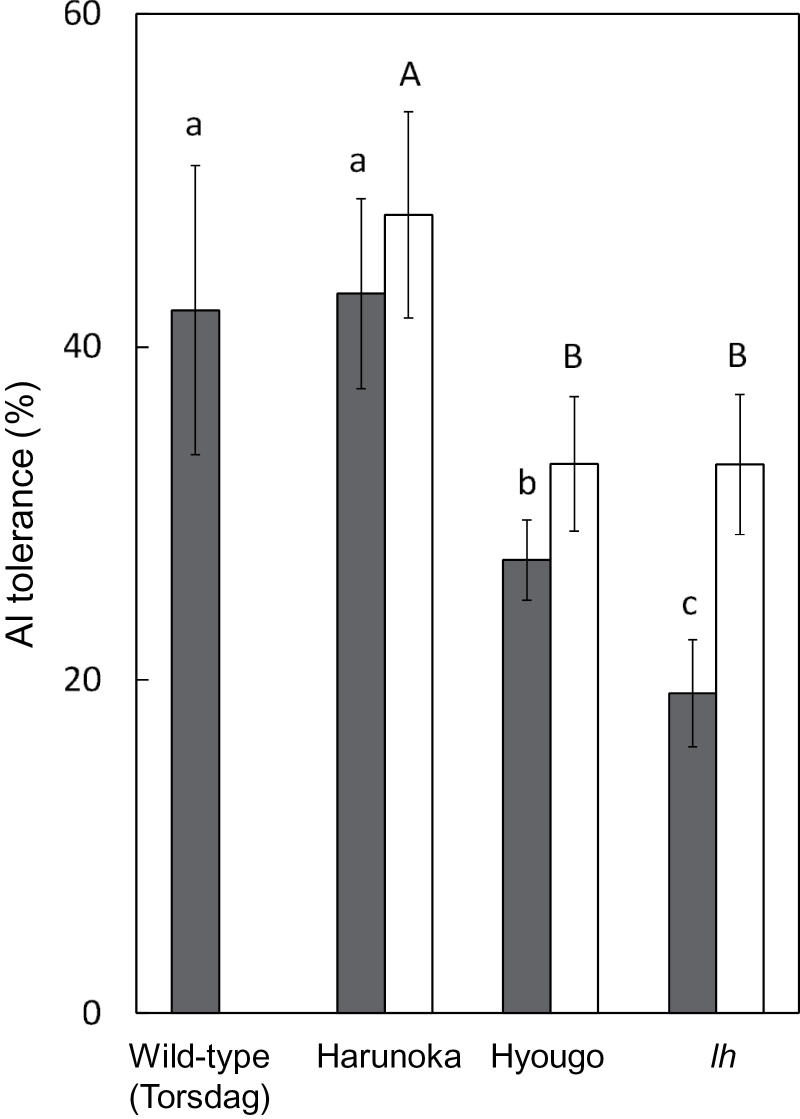
Al tolerance of pea cultivars, wild type, and GA mutants. Seedlings (5 days old) with roots ~4cm long were treated with 0.2mM CaCl_2_ with or without 20 µM AlCl_3_ (pH 4.9) for 1h (short-term experiment, open bar) or 24h (long-term experiment, closed bar). In the short-term experiment, seedlings were treated for 1h with or without Al, then with 0.2mM CaCl_2_ (pH 5.2) for 9h before evaluating root re-elongation. Al tolerance was calculated as the ratio of net primary root elongation in Al treatment to that in the control after 9h re-elongation in the short-term experiment, or 24h in the long-term experiment. Values are means of three independent replicates ± standard error. Different letters indicate significant differences among cultivars and mutants at *P* < 0.05 (Fisher’s test; Fisher, 1958).

**Fig. 2.**
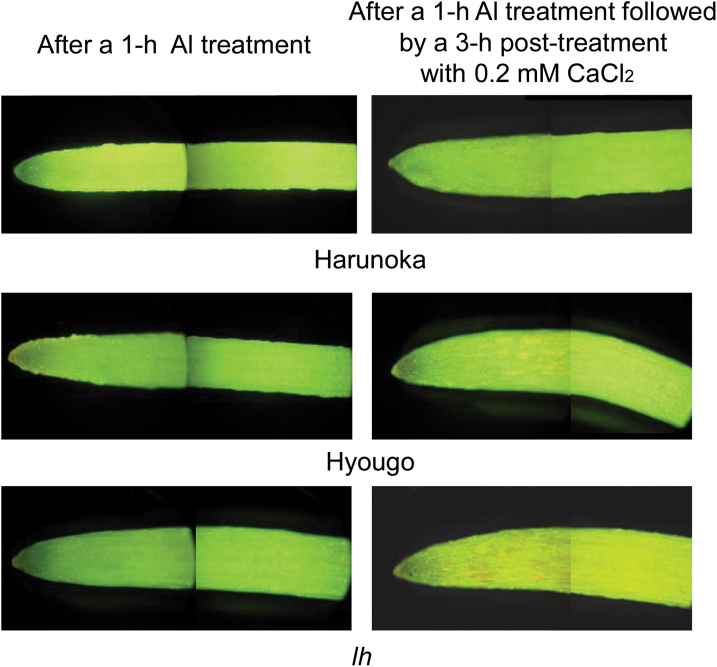
Plasma membrane permeability of the root-tip portions of pea cultivars or mutants with different degrees of Al tolerance. Roots of 5-day-old seedlings of pea cv. Harunoka (Al tolerant), cv. Hyougo (Al sensitive), or the *lh* mutant (most Al sensitive) were treated with 20 µM AlCl_3_ (pH 4.9) for 1h then with 0.2mM CaCl_2_ (pH 5.2) for 3h before measuring root re-elongation. Plasma membrane permeability was visualized by FDA-PI fluorescence microscopy after 1h of Al treatment and again after 3h of post-treatment with 0.2mM CaCl_2_. In the experiments, greenish fluorescence indicates the intactness of the plasma membrane lipid layer, and reddish-orange fluorescence indicates permeabilization of the plasma membrane lipid layer. This figure is available in colour at *JXB* online.

Using different Al treatment conditions (i.e. different durations of Al treatments), Al tolerance was compared among various cultivars, the wild type, and a mutant of pea. In one experiment, plants were exposed to toxic Al solution for 1h, and then their root re-growth over the following 9h was compared (Fig. 1, open bars). These conditions were used to assess the degree of constitutive Al tolerance in the cultivars, the wild type, and the mutant. In another experiment, roots were kept in Al toxic solution for 24h, and then their root elongation was compared (Fig. 1, closed bars). The latter conditions were used to evaluate induced Al tolerance in the cultivars and the mutant. In both conditions, the *lh* mutant and cv. Hyougo were significantly more Al sensitive than were cv. Harunoka and the wild type (Torsdag). The Al-sensitive genotypes accumulated more Al in the root tips than did Al-tolerant genotypes (Supplementary Figure S4). Additionally, the Al-sensitive genotypes showed fewer intact plasma membranes than did Al-tolerant genotypes after a 1-h Al treatment followed by a 3-h post-treatment with 0.2mM CaCl_2_ (Fig. 2).

**Fig. 3.**
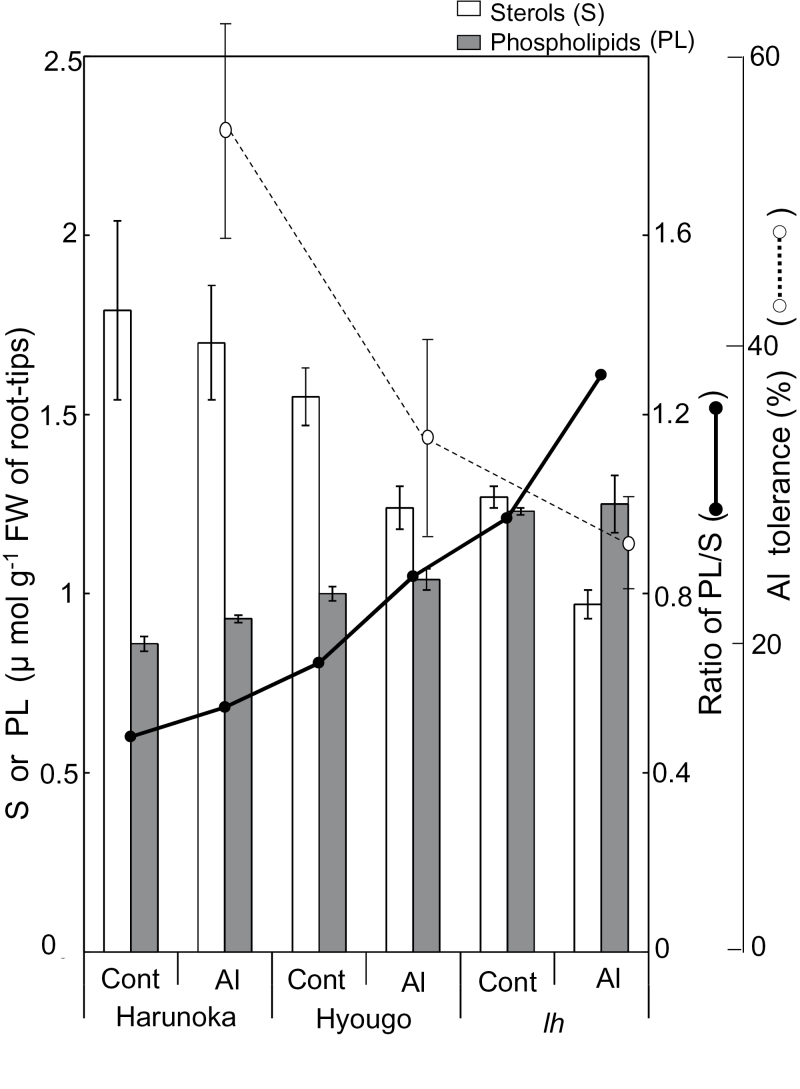
Contents of Δ^5^-sterols (S, open bar) and phospholipids (PL, closed bar), ratio of PL/S (closed circle and solid line), and Al tolerance (open circle and dotted line) of cultivars and mutants of pea. Five-day-old pea seedlings were treated with 0.2mM CaCl_2_ with or without 20 µM AlCl_3_ (pH 4.9) for 24h. S is expressed as β-sitosterol equivalents. Al tolerance was calculated as the ratio of net root elongation in Al treatment to that in the control. Values are means of three independent replicates ± standard error.

**Fig. 4.**
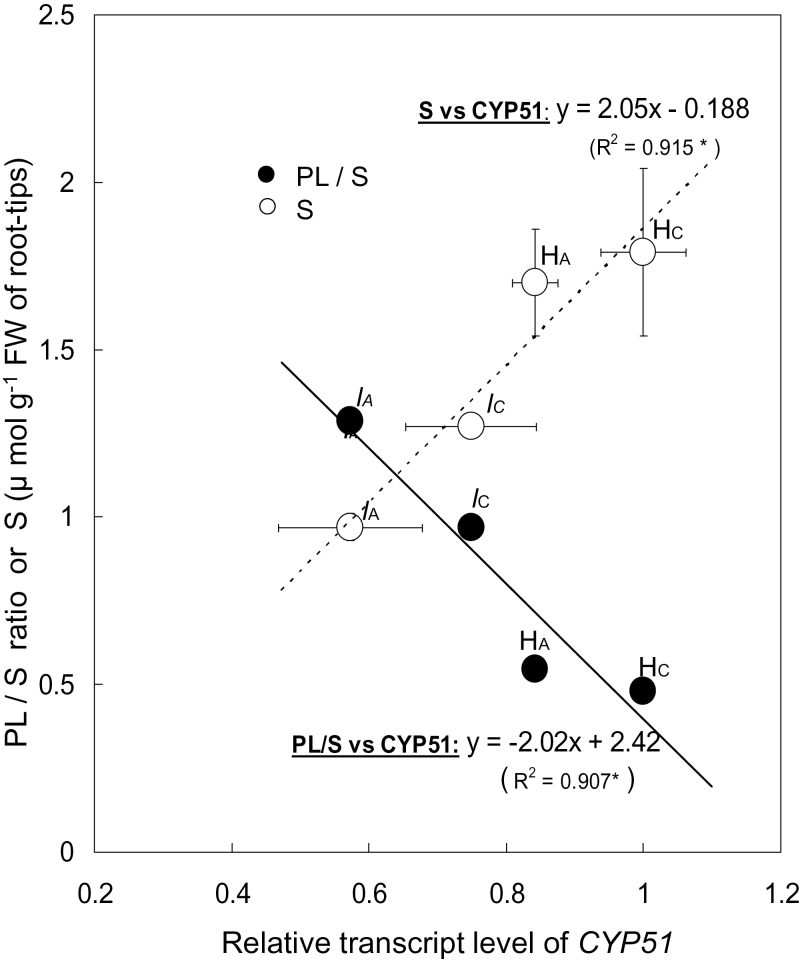
Relationship between relative transcript level of *CYP51* and phospholipid (PL)/sterol (S) ratio or sterols. Total RNA was extracted from frozen 1-cm root tips of 5-day-old pea seedlings and used for real-time qRT-PCR. The relative transcript level of *CYP51* was normalized to that of 18S rRNA (internal control). PL and S were extracted from 1-cm root tips of 5-day-old pea seedlings. The dotted line shows the relationship between S and relative transcript levels of *CYP51*. The solid line shows the relationship between relative transcript level of *CYP51* and PL/S ratio. H_C_, cv. Harunoka (control); H_A_, cv. Harunoka (Al treatment); *l*_C_, *lh* mutant (control); *l*_A_, *lh* mutant (Al treatment). Values are means of two independent replicates ± standard error.

Next, the lipid composition and expression levels of *CYP51* were evaluated in the Al-sensitive and Al-tolerant pea genotypes. The *CYP51* homologue in pea was cloned by degenerate PCR using primers designed from *CYP51* sequences in other legumes [barrel clover (*Medicago truncatula* L.) and soybean (*Glycine max* Merr.)]. A full-length cDNA, which showed high homology to orthologues in other legumes, encoded 489 amino acids. The nucleotide sequence of *PsCYP51* has been deposited in the GenBank/EMBL database [Accession number AB633330]. The *PsCYP51* transcript levels in the cultivar and the mutant were determined by qRT-PCR with specific primers. The highest concentration of sterols in the root-tip portion was in the Al-tolerant cv. Harunoka, while the highest concentration of phospholipids was in the most Al-sensitive line, the *lh* mutant (Fig. 3). In general, Al treatment decreased the sterol content (Fig. 3, open bars) and increased the phospholipid content (Fig. 3, closed bars). The highest ratio of phospholipids to sterols was in the most Al-sensitive line, the *lh* mutant. The relative level of *CYP51* mRNA in the root tip of pea was positively correlated with sterol content (*R* ^2^ = 0.915, *P* < 0.05), but negatively correlated with the ratio of phospholipids to sterols (*R* ^2^ = 0.907, *P* < 0.05) (Fig. 4). These results suggest that the sterol/phospholipid ratio, which is regulated by the biosynthesis of sterols, plays a role in determining Al tolerance.

### Effect of sterol biosynthetic inhibitor, uniconazole-P, on Al tolerance, plasma membrane permeability, and Al accumulation in several plant species

Next, the effects of the sterol biosynthetic inhibitor, uniconazole-P, on Al tolerance, plasma membrane peameability, and Al accumulation, was determined in several plant species. The Al tolerance of cultivars or lines was compared in the presence or absence of uniconazole-P, which inhibits OBT 14DM (Khan *et al.*, 2009), the product of *CYP51* (Kim *et al.*, 2005). The Al-tolerant cultivars or lines of triticale (line ST2), maize (cv. KD520), wheat (line ET8), and sorghum (cv. Super sugar) were grown in Al solutions in the presence or absence of uniconazole-P, and their root growth was compared with that of sensitive cultivars or lines of each species (triticale, line ST22; maize, cv. KD850; wheat, line ES8; sorghum, cv. Kaneko-hybrid; rice, cv. Rikuu-20). The Al-tolerant cv. Rikuu-132 was included as the reference.

**Fig. 5.**
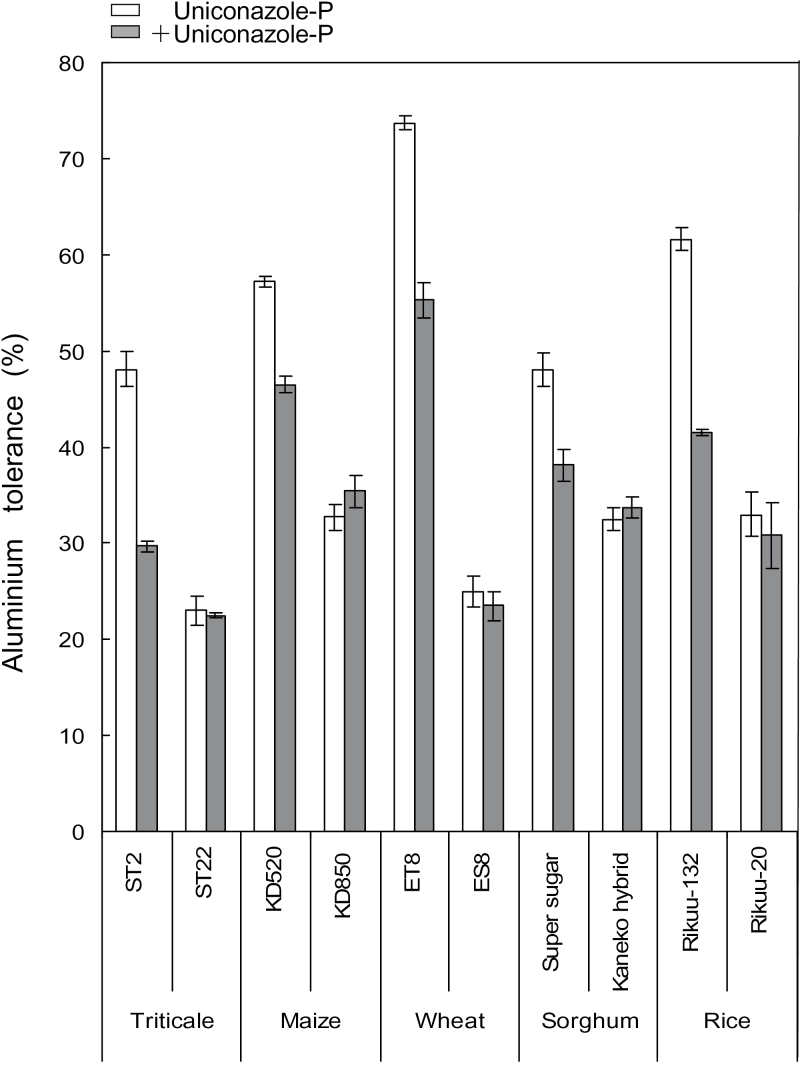
Effect of sterol biosynthetic inhibitor, uniconazole-P, on Al tolerance of several plant species, cultivars, and lines. Young seedlings with roots ~4-cm long were treated for 24h with 0.2mM CaCl_2_ ± uniconazole-P ± AlCl_3_ (maintained at pH 4.9, or pH 5.0 for sorghum only). Concentration of AlCl_3_ was 20 µM for triticale and maize, 10 µM for rice, 5 µM for wheat, and 2.5 µM for sorghum. Concentration of uniconazole-P was 0.51 µM for wheat and rice, and 1.02 µM for triticale, maize, and sorghum. Al tolerance was calculated as the ratio of root elongation of the longest root in Al treatment to that in the control. Data for rice cultivars Rikuu-132 and Rikuu-20 are from Khan *et al.* (2009). Values are means of three independent replicates ± standard error.

The effect of uniconazole-P on the Al responses of Al-tolerant cultivars and lines was evaluated using Al at a concentration that caused similar biological responses among all species (nearly 50% growth inhibition), except for wheat (30% growth inhibition). In these conditions, the sensitive cultivars and lines showed symptoms of Al toxicity, characterized by 70–80 % growth inhibition (Fig. 5). Uniconazole-P treatment suppressed the Al tolerance of all tolerant cultivars and lines, but did not affect the Al tolerance of sensitive cultivars and lines under conditions that severely inhibited root growth. Therefore, uniconazole-P affected the Al tolerance of any plant species when they were grown under moderate Al-toxicity conditions (i.e. those resulting in <50% growth inhibition.

**Fig. 6.**
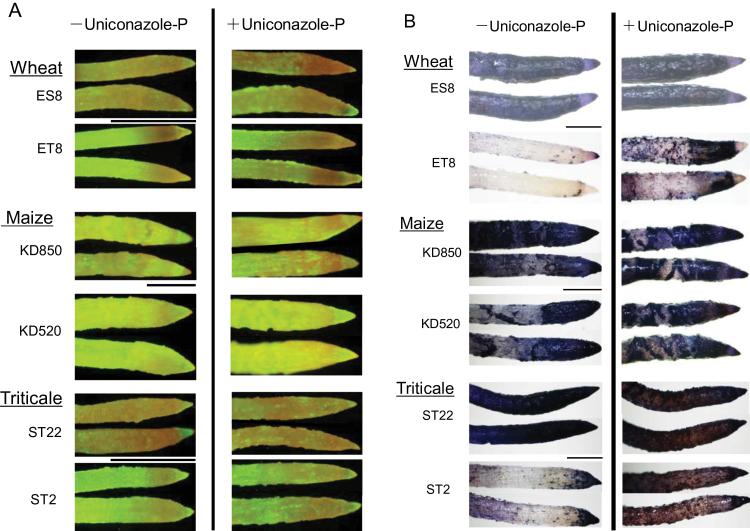
(A) Plasma membrane permeability of the root-tip portion of lines or cultivars of triticale, maize, and wheat with different degrees of Al tolerance. Plants were treated as described in Fig. 5, then roots were stained with FDA-PI to visualize plasma membrane permeability: in the experiments, reddish fluorescence indicates severe permeabilization of the plasma membrane lipid layer and greenish fluorescence indicates intactness of plasma membrane. Control seedlings (without Al) showed greenish fluorescence (data not shown). Scale bar, 1mm. (B) Al accumulation in the root-tip portion of lines or cultivars of triticale, maize, and wheat with different degrees of Al tolerance under Al treatment with or without uniconazole-P. Plants were treated as described in Fig. 5, then roots were stained with haematoxylin to visualize Al accumulation. in the experiments, a denser purple colour indicates greater Al accumulation, seen here as darker zones. Roots in control (without Al) show a whitish yellow colour, indicating little inclusion of Al (data not shown). Scale bar, 1mm. This figure is available in colour at *JXB* online.

To further analyse the effects of uniconazole-P on Al tolerance, Al accumulation and intactness of the plasma membrane were compared between cultivars or lines. Red fluorescence in FDA-PI-stained cells indicated damaged plasma membranes. The cells of tolerant cultivars and lines showed stronger red fluorescence in the presence of uniconazole-P than in its absence (Fig. 6A). Additionally, haematoxylin staining indicated that uniconazole-P enhanced Al accumulation in Al-tolerant cultivars and lines (Fig. 6B). In contrast, uniconazole-P hardly affected the intactness of the plasma membrane (Fig. 6A) or Al accumulation (Fig. 6B) in Al-sensitive cultivars and lines nor did it affect their Al tolerance (Fig. 5). These results provide further evidence that a block in the sterol biosynthetic pathway (inhibition of OBT 14DM) enhanced the Al sensitivity of Al-tolerant cultivars and lines, as a result of a loss of plasma membrane integrity and enhanced Al accumulation.

### Effect of suppression of CYP51 on Al tolerance of *Arabidopsis*

**Fig. 7.**
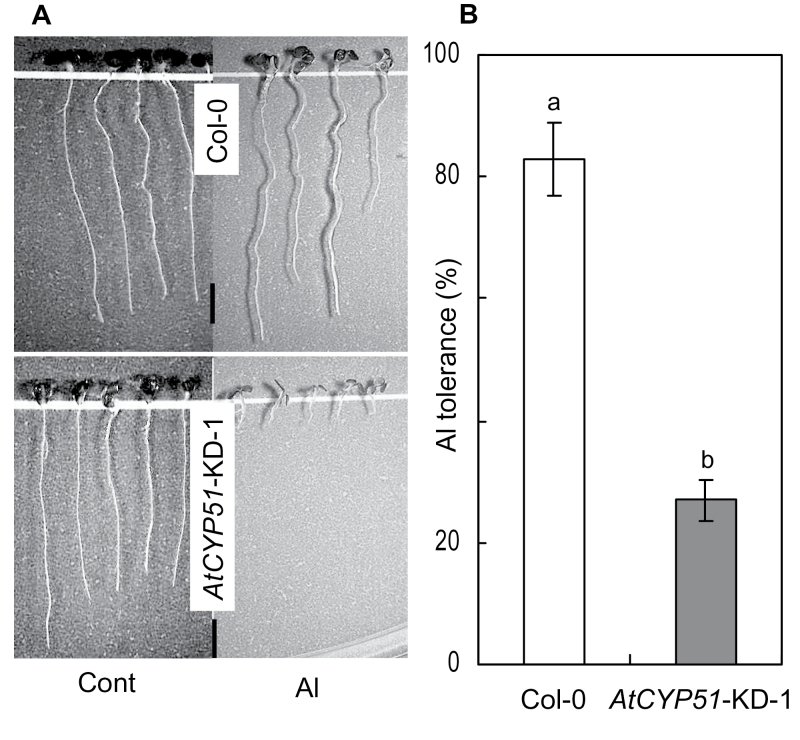
Difference in growth pattern between wild-type (Col-0) and *CYP51* knocked-down line of *Arabidopsis* (*AtCYP51*-KD-1) after Al treatment (A) and Al tolerance of Col-0 and *CYP51* knocked-down line-1 (B). Three-day-old synchronously germinated *Arabidopsis* seedlings of Col-0 and *CYP51* knocked-down line-1 were treated with or without 4 µM AlCl_3_ (pH 5.0) for 7 days. After acquiring images with a digital camera attached to a stereoscopic microscope, the length of each root in each image was measured using Image J software. Al tolerance was calculated as the ratio of root length in Al treatment to that in the control. Ten seedlings were used for each measurement. Values are means of three independent replicates ± standard error. Different lower case letters above each column indicate significant differences at a 5% level. Scale bar, 2mm.

**Fig. 8.**
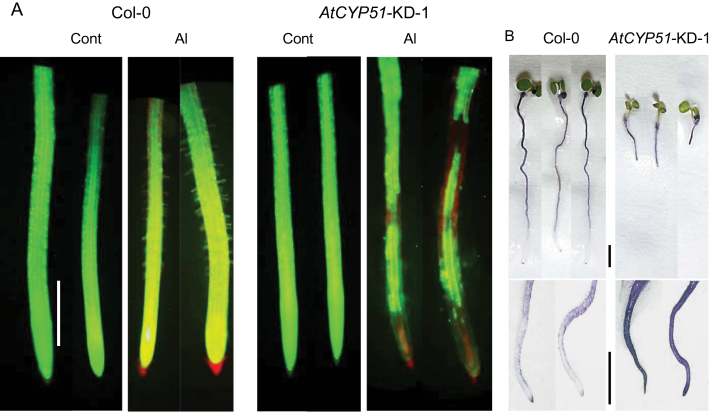
Plasma membrane permeability (A) and Al accumulation (B) in root-tip portions of *Arabidopsis* seedlings of wild-type (Col-0) and the *CYP51* knocked-down line (*CYP51*-KD-1). After 24-h treatment of *Arabidopsis* seedlings with or without 4 µM AlCl_3_ (pH 5.0), roots were stained with FDA-PI to visualize plasma membrane permeability. After a 7-day-treatment with or without 4 µM AlCl_3_ (pH 5.0), roots were stained with haematoxylin to visualize Al accumulation. In FDA-PI staining (A), in the experiments, greenish fluorescence indicates intactness of plasma membrane lipid layers and reddish fluorescence indicates permeabilization of plasma membrane lipid layers. In haematoxylin-stained tissues (B), in the experiments, denser purple staining indicates greater Al accumulation. Here this shows as darker tissue. No purple staining was observed in control *Arabidopsis* roots (data not shown). Scale bar, 1mm. This figure is available in colour at *JXB* online.

**Fig. 9.**
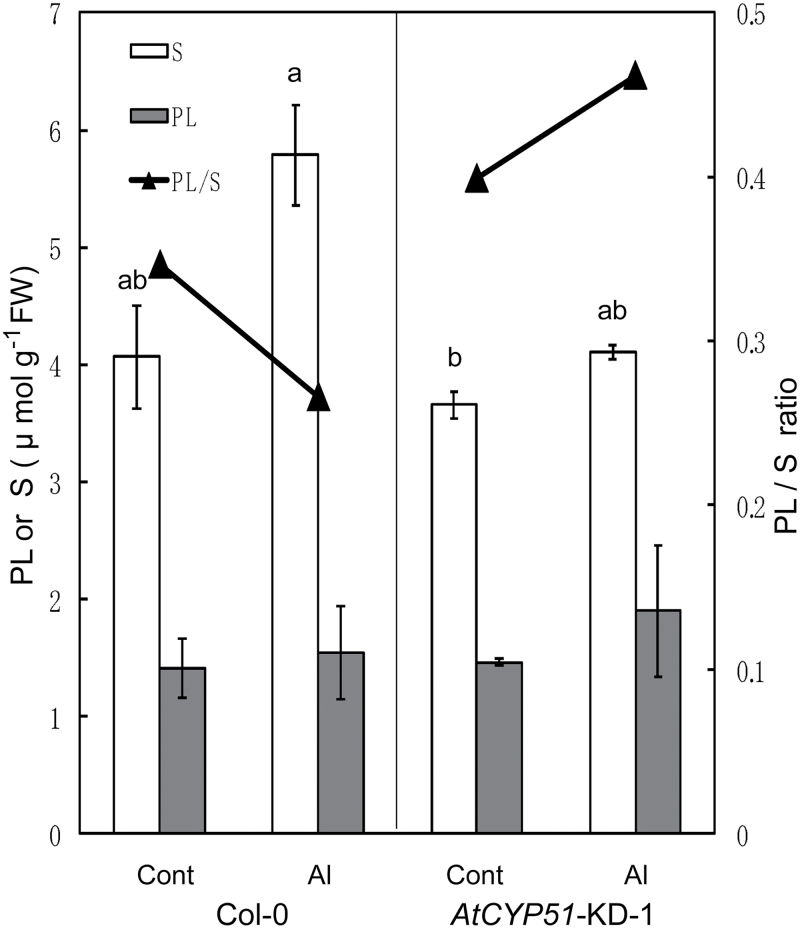
Phospholipid (PL) and sterol (S) contents in *Arabidopsis* seedlings of the wild type (Col-0) and the *CYP51* knocked-down line (*AtCYP51*-KD-1). After 7-day treatment with or without 4 µM AlCl_3_ (pH 5.0), PL and S were extracted from whole seedlings and then quantified. S is expressed as β-sitosterol equivalents. Different letters indicate significant difference at *P* < 0.05 (Fisher’s test). Values are means of three independent replicates ± standard error.

**Fig. 10.**
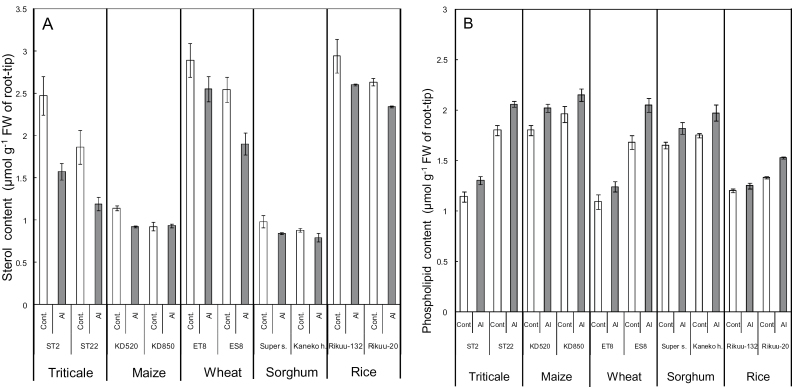
Sterol content (A) and phospholipid content (B) in the root-tip portion of several plant species treated with or without Al in the absence of uniconazole-P. Young seedlings with roots ~4cm long were treated for 24h with 0.2mM CaCl_2_ ± 2.5 µM AlCl_3_ at pH 4.9 (sorghum), and 5 µM (wheat), 10 µM (rice), or 20 µM AlCl_3_ (others) at pH 5.0. Then, 10-mm apical root samples were extracted and the contents of S and PL were determined. S is expressed as β-sitosterol equivalents. Values are means of three independent replicates ± standard error.

The results described above suggested that sterol biosynthesis, which is regulated by *CYP51*, plays a role in Al tolerance in various plant species. To further test this possibility, the Al tolerance of a transgenic *Arabidopsis* line with knocked-down *AtCYP51* expression (*AtCYP51*-KD-1; Kushiro *et al.*, 2001) was analysed. The root growth of *AtCYP51*-KD-1 was comparable to that of the wild-type Col-0 in control medium, but was inhibited in Al-containing medium (Fig. 7A, B). After 24h of Al treatment, the plasma membrane at the root-tip portion of *AtCYP51*-KD-1 was damaged (Fig. 8A) and more Al accumulated in the root tissues of *AtCYP51*-KD-1 than in those of Col-0 (Fig. 8B). The ratio of phospholipids to sterols was higher in *AtCYP51*-KD-1 than in Col-0 (Fig. 9), indicating greater negativity of the plasma membrane surface in *AtCYP51*-KD-1 than in Col-0. Because malate excretion was similar in Col-0 and *AtCYP51*-KD-1 under Al-stressed conditions (Supplementary Figure S2; Fig. 10), it is likely that the plasma membrane lipid composition had been altered as a result of suppression of *CYP51*, which enhanced the Al sensitivity of *AtCYP51*-KD-1.

## Discussion

The plasma membrane plays important roles in Al tolerance (Khan *et al.*, 2009; Maejima *et al.*, 2014). In previous studies, electrostatic computer modelling identified that {Al}^3+^ at the plasma membrane surface ({Al}^3+^_PM_) determines Al toxicity in several plant species (Wang *et al.*, 2011; Kobayashi *et al.*, 2013). Greater negativity at the plasma membrane surface increases {Al}^3+^_PM_, and phospholipids are the major source of the negative charge at the plasma membrane (Wagatsuma and Akiba., 1989; Kobayashi *et al.*, 2013). These findings indicated that a higher concentration of phospholipids in the plasma membrane increases Al toxicity. Under Pi-deprived conditions, Pi was removed from phospholipids by phosphatidate phosphohydrolases in the wild type (i.e. concomitant with increased amounts of electrically neutral galactolipids), while the phospholipid contents in the *pah1pah2* double mutant remained at a level comparable to that under Pi-sufficient conditions (Nakamura *et al.*, 2009). As a result, the double mutant showed Al sensitivity in Pi-deprived conditions, but not in Pi-sufficient conditions (Kobayashi *et al.*, 2013). Lipid composition also affects the permeability of the plasma membrane under Al-stressed conditions, which could affect Al tolerance (Khan *et al.*, 2009). The results of the present study indicate that sterol biosynthesis affected Al tolerance. This finding can be explained by the characteristics of sterols, which are electrically neutral. Because of their stereochemical structure, sterols make the plasma membrane less fluid and permeable than do several other lipid species, because the mobility of phospholipid fatty acyl chains is restricted (Hartmann, 1998).

Comparison of Al tolerance among different pea genotypes identified that the phospholipid/sterol ratio was negatively correlated with Al sensitivity (Fig. 3). The Al-sensitive phenotype was associated with greater permeability of the plasma membrane in the root-tip portion (Fig. 2) and greater Al accumulation (Supplementary Figure S4). This was correlated with suppressed expression of *CYP51*, which encodes OBT 14DM, a key enzyme in sterol biosynthesis (Kushiro *et al.*, 2001; Kim *et al.*, 2005). The Al-sensitive *lh* mutant is a low-GA mutant, because it contains a dysfunctional *PsKO1* gene. *PsKO1* encodes a cytochrome P450 in the CYP701A subfamily. *PsKO1* has *ent*-kaurene oxidase (KO) activity; that is, it catalyses the three steps of oxidation of *ent*-kaurene to *ent*-kaurenoic acid in the GA biosynthetic pathway (Davidson *et al.*, 2004). This raised the question as to whether GA biosynthesis might regulate Al tolerance in several plant species. GA has much stronger effects on hypocotyl elongation than on root elongation (Yaxley *et al.*, 2001; Tanimoto, 2002) (little retardation of root elongation in the *lh* mutant in the control; data not shown). Although the interaction between GA biosynthesis and sterol biosynthesis has not been clarified yet, there was repressed expression of *PsCYP51* in the *lh* mutant, and its phospholipid/sterol ratio was higher than that in other genotypes. These findings suggest that sterol biosynthesis, regulated by *PsCYP51*, plays a critical role in Al tolerance.

This possibility was evaluated using a combination of a reverse-genetics approach in *Arabidopsis* and chemical and biological approaches in various other plant species. Transgenic Arabidopsis with knocked-down expression of *AtCYP51*-KD-1 showed an Al-sensitive phenotype, which was characterized by greater Al accumulation and fewer intact root-tip membranes (Fig. 8). Little difference was detected in phytosterol content between Col-0 and *AtCYP51*-KD-1 under normal conditions (Fig. 9). Similar results have already been reported by Kushiro *et al.* (2001) and Kim *et al.* (2005). They suggested that these results might indicate the presence of branch pathways, which are quiet under normal conditions and might be activated by specific growth conditions and eventually produce the final end products, i.e. phytosterols. In the present experiment, knocked-down transformation and Al treatment might correspond to such specific growth conditions. Additionally, Kushiro *et al.* (2001) detected a considerably higher content of obtusifoliol in *AtCYP51*-KD [0.45 in Col-0, and 7.40 µg g^–1^ fresh weight (FW) in *AtCYP51*-KD]. Obtusifoliol is a typical abnormal sterol which is accumulated by the inhibition of OBT 14DM (Burden *et al.*, 1987), and is considered to occupy greater van der Waals volume, which induces greater plasma membrane permeability (Khan *et al.,* 2009). Recently, a unique but less productive sterol biosynthetic pathway through lanosterol (which had been considered to be specific for fungi and mammals) was found in dicotyledonous plants in response to several stimuli (Kolesnikova *et al.*, 2006; Suzuki *et al.*, 2006). Further explanation cannot be given at present because of the lack of detailed knowledge on the whole sterol biosynthetic pathway in plants. Uniconazole-P suppressed Al tolerance in the Al-tolerant cultivars/lines of various plant species. Uniconazole-P is a triazole-type fungicide that inhibits OBT 14DM, a key enzyme in the post-squalene sterol biosynthetic pathway (Benveniste, 1986). In the presence of Al at a concentration inducing 20–40 % growth inhibition of tolerant cultivars/lines, uniconazole-P suppressed the Al tolerance of Al-tolerant cultivars/lines (Fig. 5). This concentration of Al almost completely inhibited root growth in Al-sensitive cultivars/lines. Each concentration of uniconazole-P (0.51 µM for wheat and rice, and 1.0 µM for triticale, maize, and sorghum) had little effect on normal root elongation and plasma membrane permeability in the control solution (data not shown). The universal effect of uniconazole-P on different plant species suggested that suppression of Al tolerance resulted from the effect of this chemical in modifying plasma membrane lipid composition, and not from its interference with different Al-tolerance mechanisms in each plant species.

The results of these experiments strongly suggest that sterol biosynthesis plays an important role in the Al tolerance of plants. Previous studies on sterol inhibitors including uniconazole-P have suggested that inhibition of OBT 14DM has multiple effects on the composition of plasma membrane lipids (Khan *et al.*, 2009). Inhibition of OBT 14DM increased phospholipid contents, resulting in greater electrical negativity at the plasma membrane surface (Kobayashi *et al.*, 2013), and may also have increased the content of abnormal sterols, thereby increasing plasma membrane permeability (Kushiro *et al.*, 2001; Khan *et al.*, 2009). These changes could enhance Al sensitivity as a result of an increased Al concentration at the plasma membrane surface and increased Al permeability through the plasma membrane. Based on these findings, the suppression of *CYP51* expression under conditions in which Al inhibits root growth could further enhance Al toxicity.

In control conditions without Al, the root-tip portions of all Al-tolerant cultivars and lines used in these experiments contained a higher sterol content and a lower phospholipid content than those of the Al-sensitive ones (Fig. 10). Additionally, Al treatment decreased the sterol content and increased the phospholipid contents. To date, there have been no reports on the reverse cross-talk between sterol content and phospholipid content in the root-tip portion (Supplementary Figure S4). Several nonspecific lipid transfer proteins (nsLTPs) transferring phospholipids, glycolipids, fatty acids, and steroids among membranes with wide-ranging binding affinities (Cheng *et al.*, 2004), on lipid-transfer proteins (Peretti *et al.*, 2008), or on other proteins (Jouhet *et al.*, 2007), may be one of the related candidates for the reverse cross-talk. Plasma membrane lipids play other roles such as stabilization of raft structures, which are important to maintain stress recognition and signal transduction through plasma membrane proteins (Lynch and Dunn, 2004). Further research should be conducted to uncover more details of the complex roles of plasma membrane lipids in Al tolerance.

## Supplementary material

Supplementary data can be found at *JXB* online.

Supplementary Figure S1. Difference in growth pattern of *Arabidopsis* between wild-type (Col-0) and transformant (*AtCYP51*-KD-1).

Supplementary Figure S2. Relative transcript levels of *AtCYP51* in wild-type Col-0 and *CYP51* knocked-down lines of *Arabidopsis*.

Supplementary Figure S3. Malate release from *Arabidopsis* roots.

Supplementary Figure S4. Al accumulation in the root-tip portion of pea.

Supplementary Figure S5. Relationship between sterol content and phospholipid content in the root-tip portion of pea and rice.

## Funding

This work was supported by Grants-in-Aid for Scientific Research (A) and (B) (Nos 18208008, 23380041) from the Japan Society for the Promotion of Science (JSPS).

## Supplementary Material

Supplementary Data
