# Supplementary material for: Higher sterol content regulated by CYP51 with concomitant lower phospholipid content in membranes is a common strategy for aluminium tolerance in several plant species
Source: J Exp Bot. 2014 Nov 21;66(3):907–18. doi: 10.1093/jxb/eru455 (PMC4321553; doi:10.1093/jxb/eru455)
Supplement: Supplementary Data [file supp_66_3_907__index.html]

Higher sterol content regulated by CYP51 with concomitant lower phospholipid contents in membranes is a common strategy for aluminium tolerance in several plant species — Higher sterol content regulated by CYP51 with concomitant lower phospholipid content in membranes is a common strategy for aluminium tolerance in several plant species — Supplementary Data 

# Higher sterol content regulated by *CYP51* with concomitant lower phospholipid content in membranes is a common strategy for aluminium tolerance in several plant species

## Supplementary Data

Data files

**Files in this Data Supplement:**

- Supplementary Data - Supplementary Data
