# Supplementary material for: Higher sterol content regulated by CYP51 with concomitant lower phospholipid content in membranes is a common strategy for aluminium tolerance in several plant species
Source: J Exp Bot. 2014 Nov 21;66(3):907–18. doi: 10.1093/jxb/eru455 (PMC4321553; doi:10.1093/jxb/eru455)
Supplement: Supplementary Data [file supp_eru455_jexbot134114_file001.pdf]

SUPPLEMENTARY MATERIAL

Higher sterols content regulated by *CYP51* with concomitant lower phospholipids content in membranes is a common strategy for aluminium tolerance in several plant species

T Wagatsuma, MSH Khan, T Watanabe, E Maejima, H Sekimoto, T Yokota, T Nakano, T Toyomasu, K Tawaraya, H Koyama, M Uemura, S Ishikawa, T Ikka, A Ishikawa, T Kawamura, S Murakami, N Ueki, A Umetsu, T Kannari

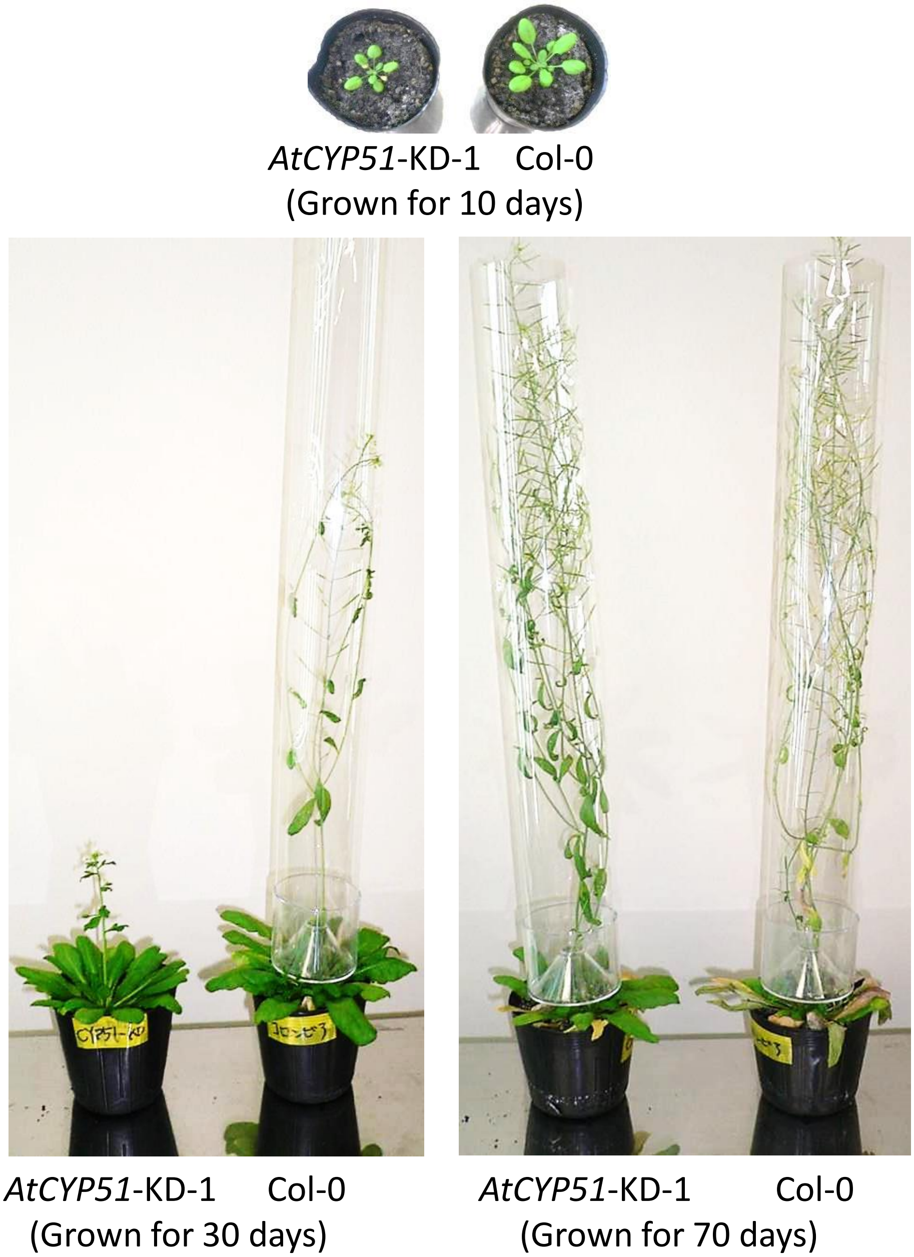

Fig. S1. Difference in growth pattern of Arabidopsis between wild-type (Col-0) and transformant (*AtCYP51*-KD-1) under normal growth conditions. Seedlings were watered for 1 week, and then grown for 3 months individually under transparent plastic cylinders to avoid cross-pollination. Plants were fertilised once weekly with 1/1000 dilution of HYPONeX nutrient solution. Pictures were taken after 10, 30, and 70 days from the start of growth. *AtCYP51*-KD-1 seedling showed a semi-dwarf phenotype in the early growth stage and a longer life span than that of wild-type, as described in Kushiro *et al*. (2001).

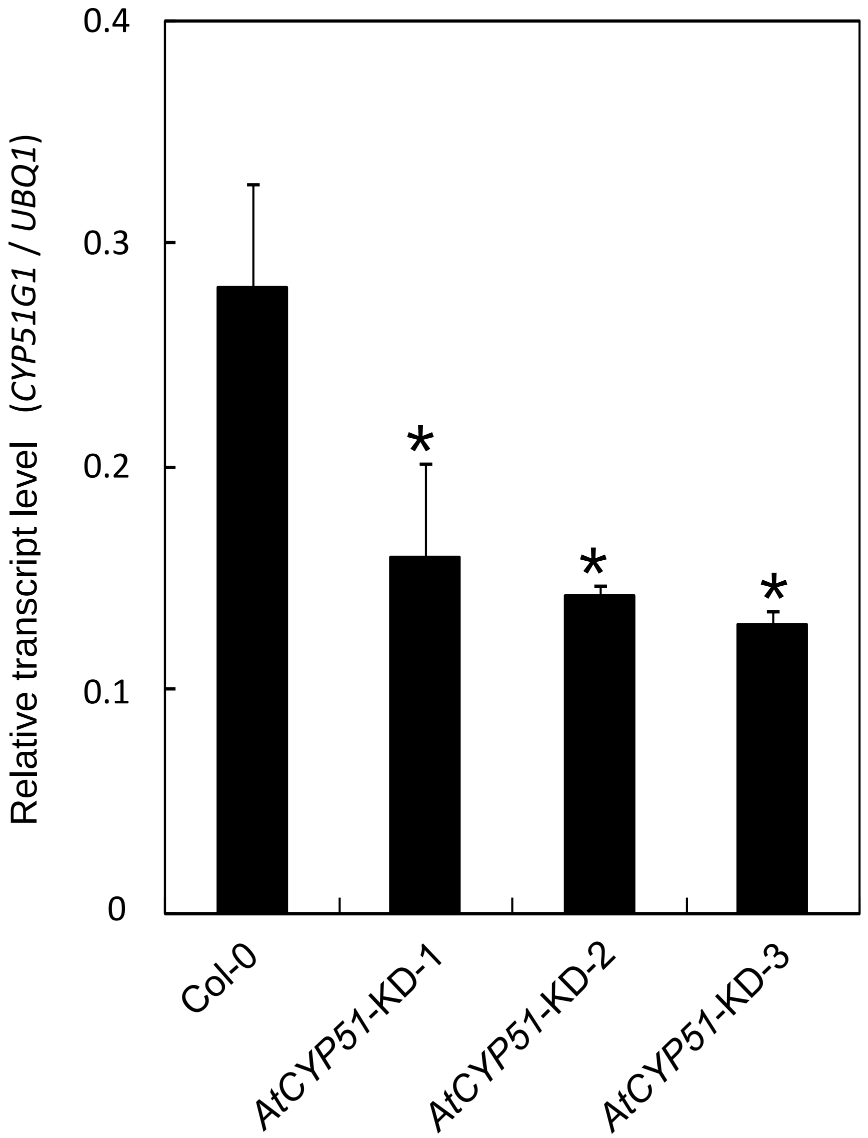

Fig. S2. Relative transcript levels of wild-type Col-0 and *CYP51G1* knocked-down lines of Arabidopsis. Seedlings were grown hydroponically in modified MGRL medium for 1 week in the presence of 4 µM $AlCl_3$. Total RNA was extracted from roots. Transcript levels of *CYP51* and *UBQ1* were quantified by real-time PCR. Values are means of three independent replicates ± standard error. Asterisk shows significant difference between Col-0 and *CYP51G1* knocked-down lines (Fisher's test). Transcript levels of *AtCYP51* were significantly lower in all three *AtCYP51*-KD lines than in wild-type.

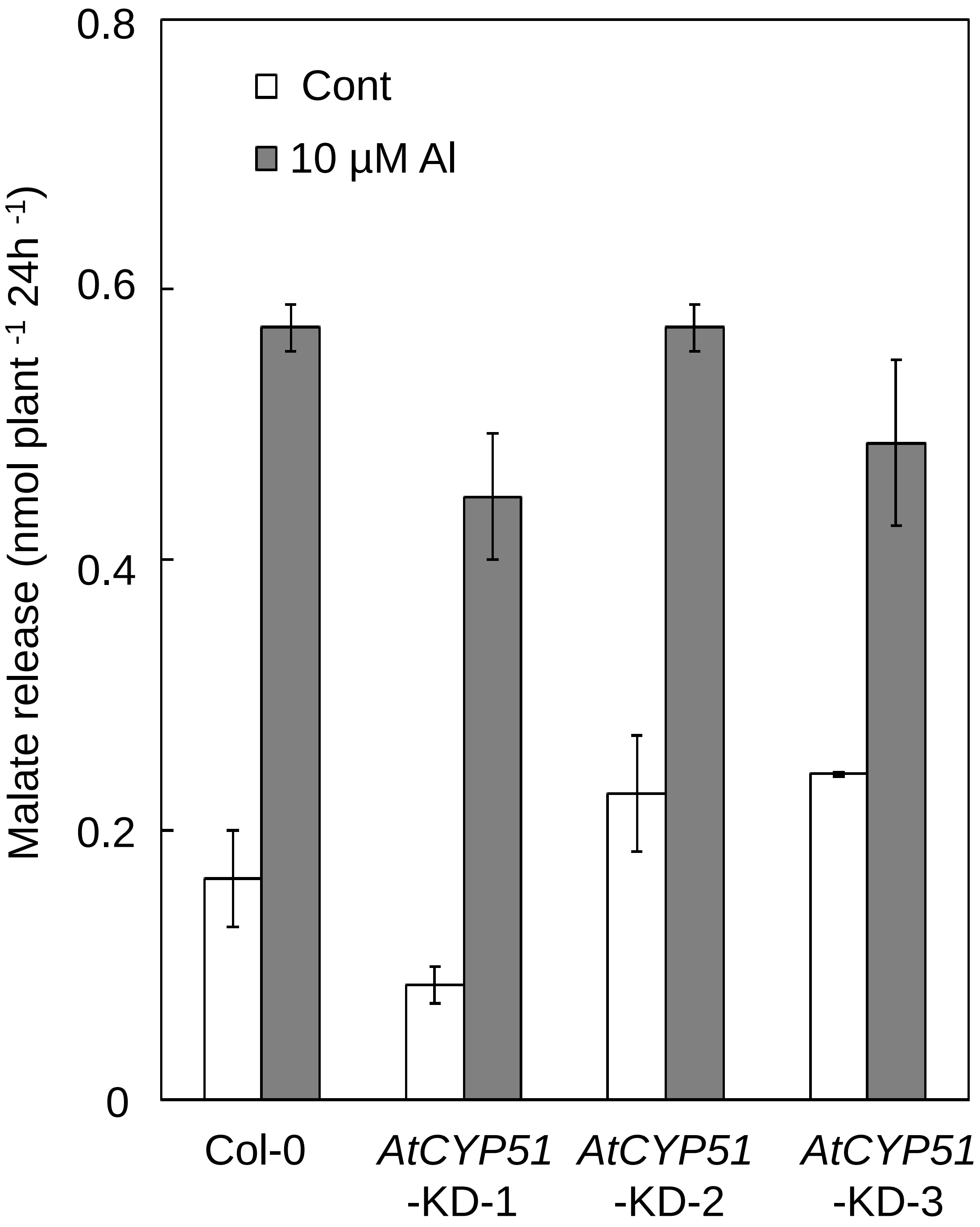

Fig. S3. Malate release from Arabidopsis roots. Analysis of malate from Arabidopsis was carried out as described by Kobayashi *et al*. (2007). Roots of wild-type (Col-0) and three *CYP51* knocked-down lines (*CYP51*-KD line-1, -2 and -3) of Arabidopsis were exposed to 0.2 mM $CaCl_2$ with or without 10 µM $AlCl_3$ (pH 5.0) for 24 h. Values are means of three independent replicates ± standard error.

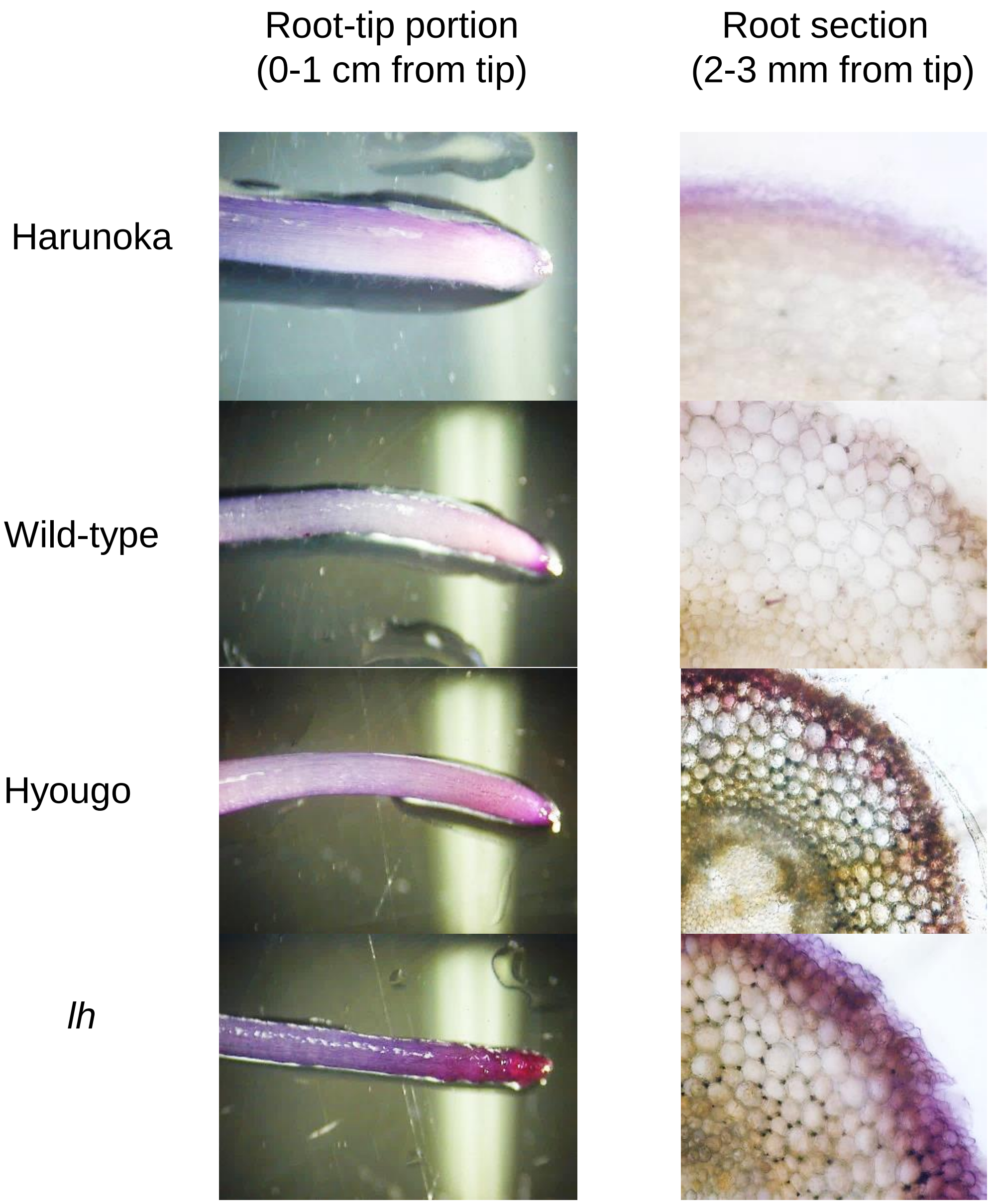

Fig. S4. Al accumulation in root-tip portion of pea. Roots of 5-day-old pea seedlings were treated for 1 h with or without 20 µM $AlCl_3$ (pH 4.9). After Al treatment, roots were stained with haematoxylin for 15 min (Polle *et al.*, 1978). Al accumulation in tip portion was observed by stereoscopy and Al distribution in section of root 2 -3 mm from apex was observed by light microscopy. Denser purple colour indicates greater Al accumulation.

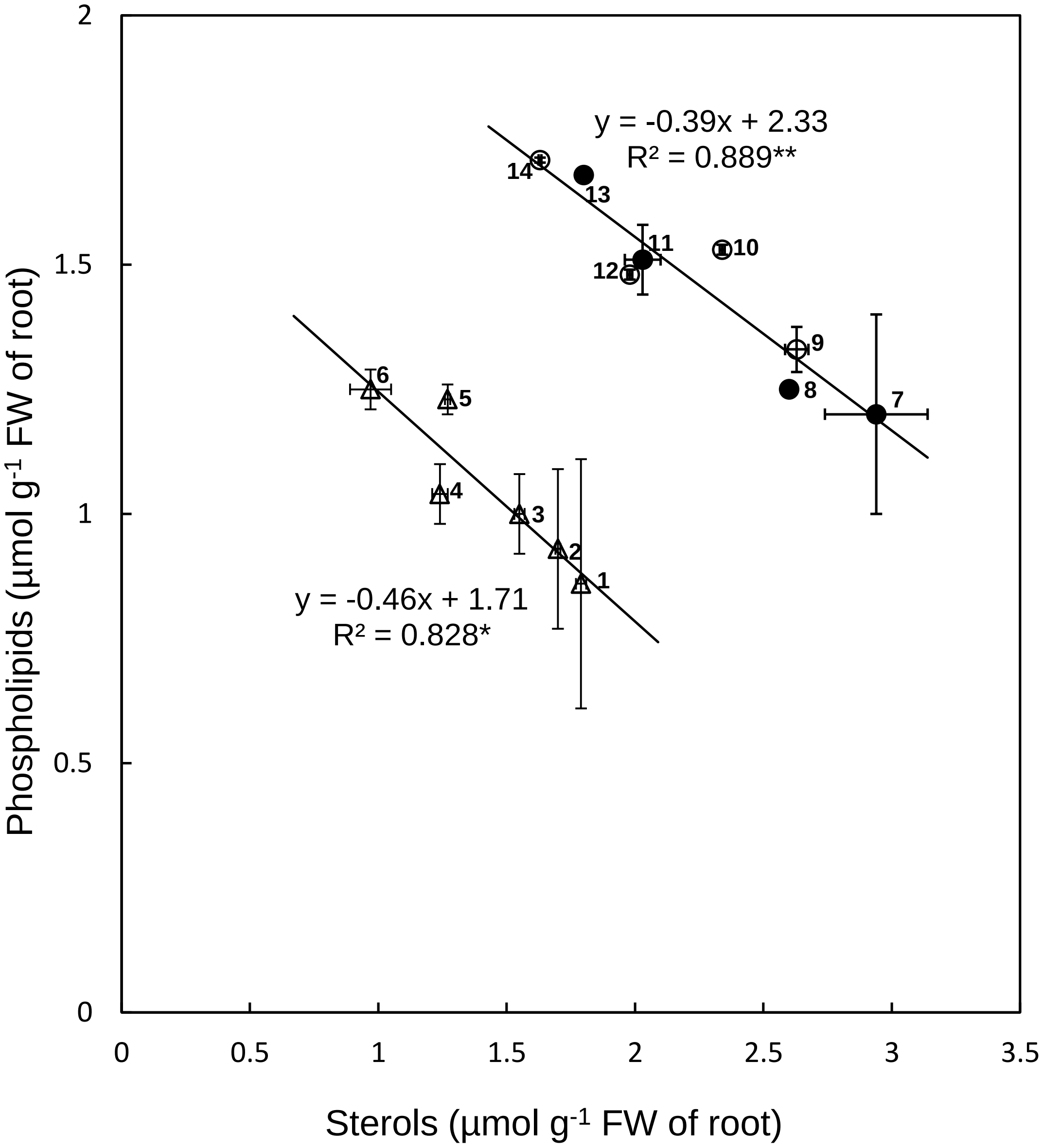

Fig. S5. Relationship between sterol content and phospholipid contents in root-tip portion of pea (cv. Harunoka, cv. Hyougo, *lh* mutant; control, Al treatments) (triangles) and rice (cv. Rikuu-132, cv. Rikuu-20; control, Al, uniconazole-P, Al + uniconazole-P treatments) (circles). Harunoka, cont (1); Harunoka, Al (2); Hyougo, cont (3); Hyougo, Al (4); *lh*, cont (5); *lh*, Al (6); Rikuu-132, cont (7); Rikuu-132, Al (8); Rikuu-20, cont (9); Rikuu-20, Al (10); Rikuu-132, uniconazole-P (11); Rikuu-20, uniconazole-P (12); Rikuu-132, Al + uniconazole-P (13); Rikuu-20, Al + uniconazole-P (14). Data for pea are same as those in Fig. 3. Data for rice are from Fig. 4 in Khan *et al*. (2009).
